# A Map of Transcriptomic Signatures of Different Brain Areas in Alzheimer’s Disease

**DOI:** 10.3390/ijms252011117

**Published:** 2024-10-16

**Authors:** Riccardo Rocco Ferrari, Valentina Fantini, Maria Garofalo, Rosalinda Di Gerlando, Francesca Dragoni, Bartolo Rizzo, Erica Spina, Michele Rossi, Chiara Calatozzolo, Xhulja Profka, Mauro Ceroni, Antonio Guaita, Annalisa Davin, Stella Gagliardi, Tino Emanuele Poloni

**Affiliations:** 1 Department of Brain and Behavioral Sciences, University of Pavia, Viale Golgi 19, 27100 Pavia, Italy; 2 Laboratory of Neurobiology and Neurogenetics, Golgi Cenci Foundation, Corso San Martino 10, 20081 Abbiategrasso, Italy; valentina.fantini92@gmail.com (V.F.); e.spina@golgicenci.it (E.S.); a.guaita@golgicenci.it (A.G.); 3 Laboratory of Translational Research, Azienda USL-IRCCS of Reggio Emilia, Viale Risorgimento 80, 42123 Reggio Emilia, Italy; 4 Molecular Biology and Transcriptomic Unit, IRCCS Mondino Foundation, Via Mondino 2, 27100 Pavia, Italy; garofalomaria.bio@gmail.com (M.G.); rosalinda.digerlando01@universitadipavia.it (R.D.G.); francesca.dragoni@mondino.it (F.D.); bartolo.rizzo@mondino.it (B.R.); stella.gagliardi@mondino.it (S.G.); 5 Department of Biology and Biotechnology “L. Spallanzani”, University of Pavia, Via Adolfo Ferrata 9, 27100 Pavia, Italy; 6 Unity of Biostatistics, Golgi Cenci Foundation, Corso San Martino 10, 20081 Abbiategrasso, Italy; m.rossi@golgicenci.it; 7 Department of Neurology and Neuropathology, Golgi Cenci Foundation, Corso San Martino 10, 20081 Abbiategrasso, Italy; c.calatozzolo@golgicenci.it (C.C.); x.profka@golgicenci.it (X.P.); mauro.ceroni@unipv.it (M.C.); e.poloni@golgicenci.it (T.E.P.); 8 Department of Rehabilitation, ASP Golgi-Redaelli, Piazza E. Samek Lodovici 5, 20081 Abbiategrasso, Italy

**Keywords:** Alzheimer’s disease, transcriptomics, human brain samples, different brain regions, molecular map

## Abstract

Alzheimer’s disease (AD) is a neurodegenerative disorder that progressively involves brain regions with an often-predictable pattern. Damage to the brain appears to spread and worsen with time, but the molecular mechanisms underlying the region-specific distribution of AD pathology at different stages of the disease are still under-investigated. In this study, a whole-transcriptome analysis was carried out on brain samples from the hippocampus (HI), temporal and parietal cortices (TC and PC, respectively), cingulate cortex (CG), and substantia nigra (SN) of six subjects with a definite AD diagnosis and three healthy age-matched controls in duplicate. The transcriptomic results showed a greater number of differentially expressed genes (DEGs) in the TC (1571) and CG (1210) and a smaller number of DEGs in the HI (206), PC (109), and SN (60). Furthermore, the GSEA showed a difference between the group of brain areas affected early (HI and TC) and the group of areas that were subsequently involved (PC, CG, and SN). Notably, in the HI and TC, there was a significant downregulation of shared DEGs primarily involved in synaptic transmission, while in the PC, CG, and SN, there was a significant downregulation of genes primarily involved in protein folding and trafficking. The course of AD could follow a definite time- and severity-related pattern that arises from protein misfolding, as observed in the PC, CG, and SN, and leads to synaptic impairment, as observed in the HI and TC. Therefore, a map of the molecular and biological processes involved in AD pathogenesis may be traced. This could aid in the discovery of novel biological targets in order to develop effective and well-timed therapeutic approaches.

## 1. Introduction

Alzheimer’s disease (AD) is a neurodegenerative disorder that is considered the main cause of dementia, and its prevalence is expected to double in Europe and triple worldwide by 2050 [1]. The clinical manifestations of AD are due to the progressive dysfunction of synapses and neuronal circuits, causing gradual impairment in all cognitive domains [2]. In the brain, the pathology of AD is represented by the extracellular aggregation of amyloid beta (Aβ) peptides in amyloid plaques and the intraneuronal aggregation of hyperphosphorylated Tau protein (pTau) in neurofibrillary tangles (NFTs) and threads. The multifocal coupling of Aβ plaques and NFTs in the cerebral cortex generates so-called neuritic plaques (NPs), which are the neuropathological hallmark of AD. The clinical evolution of AD reflects the propagation of synaptic failure, which is accompanied or followed by the propagation of the pathology across various brain areas; typically, AD pathology starts in the parahippocampal, entorhinal, and hippocampal zones and propagates to the temporo-parietal cortex and, in some cases, the whole neocortex [3,4,5,6]. However, it is important to state that a certain degree of AD pathology, as well as brain atrophy, is often observed in cognitive healthy elderly [7]. Considering that the newest anti-amyloid treatments resulted in no clinical changes that were perceived by patients and caregivers, it is becoming increasingly clear that the mechanisms that cause some subjects to develop AD and some to maintain cognitive function are quite complex and multifactorial, and they are not sufficiently explained by the amyloid cascade theory [8,9,10]. In this regard, different hypotheses about the etiology of AD have been proposed, ranging from the so-called “mitochondrial cascade hypothesis” [11] and neuroinflammation [12] to defective proteostasis, which is also strictly related to synapse failure [13]. Thus, it is becoming clear that AD’s etiology is highly heterogeneous and requires new methodological approaches; genetic and transcriptomic investigations could be winning strategies for understanding the pathogenesis of AD. Transcriptomic analyses, in particular, are crucial for obtaining information about differentially expressed genes (DEGs) and alternative gene expression regulation mechanisms that were already observed to impact neurodegeneration [14]. Moreover, RNA-seq data could be used to build functional interaction networks and to obtain additional information about the active role of specific genes in a specific cellular model [15] or pathological context [16]. There are few transcriptomic studies focused on AD as a whole-brain pathology. Generally, analyses were performed on the most affected areas, such as the hippocampus and entorhinal cortex [14,17], considered specific aspects of the pathology [18,19], or mainly focused on a subtype of deregulated RNA, such as miRNA and lncRNA [20,21]. There are also studies focusing on less affected areas, such as the primary visual cortex, that aim to investigate AD without the influence of other collateral aspects of the pathology [22]. However, even if of great interest, no information about the differences between brain regions among the selected subjects has been reported. Finally, we did not find any other research aimed at tracing a molecular map of the spread of AD in the brain by simultaneously analyzing and comparing the transcriptomic profiles of different brain regions or, moreover, research comparing AD with CTRL subjects.

Considering the above-mentioned propagation pattern of AD, we performed a whole-transcriptome analysis on human post-mortem brain tissue samples derived from the hippocampus (HI), temporal cortex (TC), parietal cortex (PC), cingulate gyrus (CG), and substantia nigra (SN) of AD subjects and compared them with those of subjects without dementia. In order to increase the value of this study, we used post-mortem samples from a cohort of elderly subjects that we had monitored since 2010, when they were enrolled in the InveCe.Ab study [23], a population-based longitudinal study that is currently ongoing in Abbiategrasso. Therefore, our first goal was to define the “molecular signature” of AD in each brain region considered. To gain insight into the biological role of the observed deregulated genes (DEGs) and to build region-specific functional interaction networks, a gene set enrichment analysis (GSEA) was performed on the total number of DEGs and then only on the deregulated mRNAs. Our second objective was to switch from a region-specific molecular signature to a whole-brain molecular map of the spread of AD. To do so, we compared the functional enrichment terms of every brain region, an analysis that resulted in the spontaneous clustering of specific brain regions based on two well-defined groups of functional enrichment networks. This clustering may have revealed a shared deregulation pattern, and it was hypothesized that it reflected the propagation pattern of AD. This would allow us to work backwards and identify specific coding DEGs whose deregulation could be related to the etiology of AD.

## 2. Results

The results are reported according to the two main research questions relative to the molecular signature of AD in each brain region and the molecular map of AD’s spread in the brain.

### 2.1. AD Subjects Showed Different Transcriptomic Profiles from Those of the CTRL Subjects

#### 2.1.1. Hippocampus—HI

**Figure 1:**
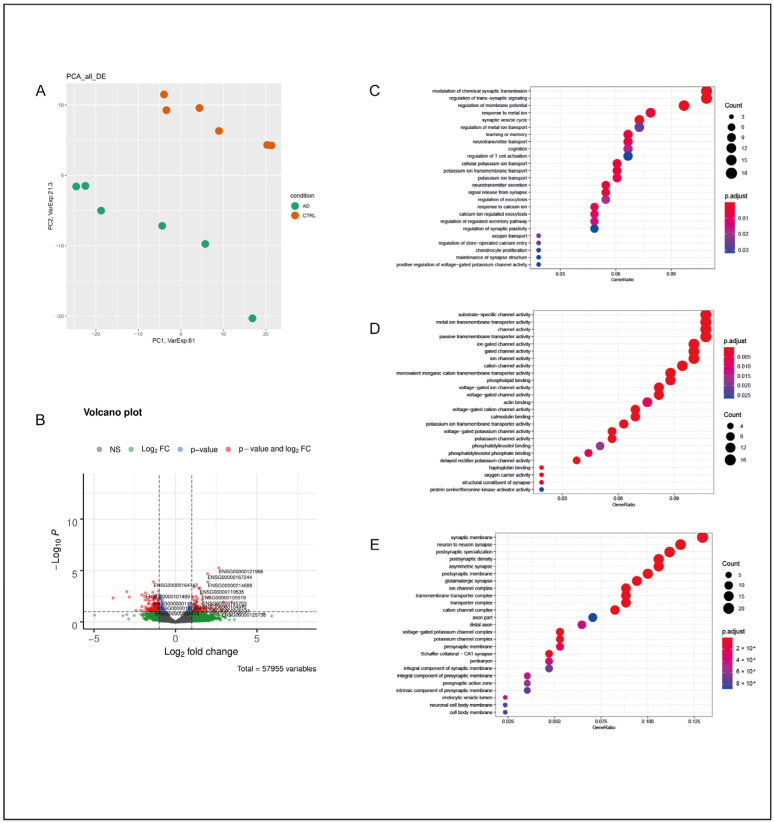
Hippocampus. (**A**) PCA of DEGs in the HI of AD subjects in comparison with those of the CTRL subjects; AD subjects and CTRL subjects were separated into two distinct groups, suggesting a substantial difference between the two groups from a statistical point of view. (**B**) Volcano plot of DEGs between AD and CTRL subjects. Genes were plotted in order to emphasize both statistical significance (*p*-value) and magnitude of change (Log_2_ fold change). Genes whose deregulation was the most statistically significant and that had a large fold change are represented with red dots. (**C**) GO-enriched terms in the HI of AD vs. CTRL for biological processes, molecular functions (**D**), and cellular components (**E**). The dots in each category may vary in color and dimension; a color that tends toward red indicates a higher statistical significance, while large dots indicate a higher number of genes of that specific enrichment term that were deregulated in the analyzed sample in comparison with the original GO classification.

In the HI of AD subjects, the total number of DEGs was 206, with the majority of them being of the protein-coding class (177) and downregulated (110). Their classification is reported in Table 1. A principal component analysis (PCA) division based on the expression levels of all deregulated mRNAs and lncRNAs in the AD and CTRL groups resulted in a consistent separation between the two groups of subjects and is represented in Figure 1A.

A GO term enrichment analysis was carried out for DEGs in brain tissue from the HI of AD subjects compared with that from CTRL subjects [24,25]. For the HI of AD subjects, the GO enrichment analysis resulted in a significant deregulation of processes mainly related to synaptic transmission, such as the “modulation of chemical synaptic transmission” and the “regulation of trans-synaptic signaling” (Figure 1C). Thus, alterations in gated and ion channels and transporters were observed, together with the involvement of several cellular components mainly related to synaptic functionality and, in general, neuron-to-neuron interactions (Figure 1D,E). A STRING analysis was performed on 96 out of 177 protein-coding DEGs with an adjusted *p* value of ≤0.05. In the HI, a total of four local network clusters were found, showing a functional correlation between DEGs that were exclusively involved in voltage-gated potassium and sodium- and calcium-activated potassium channel activity (Supplementary Table S3). A similar outcome was obtained when considering the top five GO terms in the STRING analysis for biological processes, molecular functions, and cellular components of the functional enrichment analysis. The deregulation of processes related to the synaptic vesicle cycle and synaptic plasticity was observed, with a strong involvement of the presynaptic active zone (AZ) (Supplementary Table S4), a result consistent with the GO analyses performed on all DEGs. Interestingly, the DEGs of the top five STRING GO terms of the functional enrichment analysis resulted in downregulation in the HI of AD subjects, except for *LTF*, which encodes for the protein Lactoferrin (Lf).

#### 2.1.2. Temporal Cortex—TC

**Figure 2:**
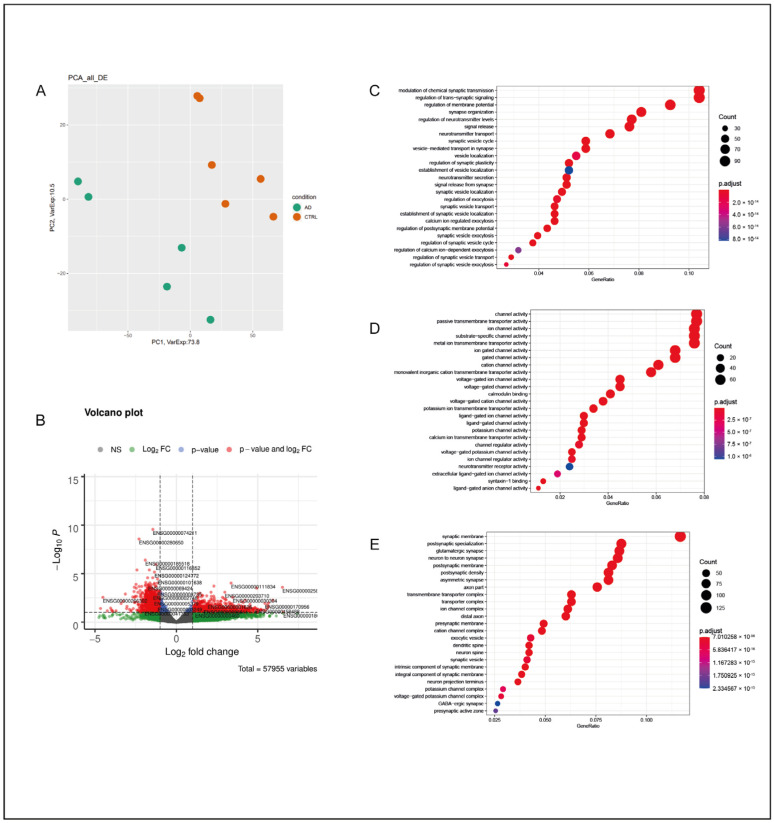
Temporal cortex. (**A**) PCA of DEGs in the TC of AD subjects in comparison with CTRL subjects. AD subjects and CTRL subjects were separated into two distinct groups, suggesting a substantial difference between the two groups from a statistical point of view. (**B**) Volcano plot of DEGs between AD and CTRL subjects. Genes were plotted in order to emphasize both statistical significance (*p*-value) and magnitude of change (Log_2_ fold change). Genes whose deregulation was most statistically significant and that had a large fold change are represented by red dots; (**C**) GO-enriched terms in the TC of AD vs. CTRL for biological processes, molecular functions (**D**), and cellular components (**E**). The dots in each category may vary in color and dimension; a color that tends toward red indicates greater statistical significance, while large dots indicate a higher number of genes of that specific enrichment term that were deregulated in the analyzed sample in comparison with the original GO classification.

In the TC of AD subjects, the total number of DEGs was 1571, with the majority of them being protein-coding (1152) and downregulated (781). Their classification is reported in Table 1. The PCA division based on the expression levels of all deregulated mRNAs and lncRNAs in the TC of AD subjects and CTRL subjects resulted in a defined separation between the groups of subjects, and this is presented in Figure 2A. One AD sample is missing because it failed RNA sequencing, and no more samples were available.

For the TC of AD subjects, the GO biological process analysis resulted in a significant deregulation of processes related to synaptic functionality and transmission (Figure 2C). Thus, a strong alteration in the activity of several families of ion channels was observed (Figure 2D), together with the involvement of cellular components (Figure 2E) related to pre- and post-synaptic membranes and neuronal components such as axons, neuron spines, and neurites. A STRING analysis was then performed on 727 out of 1152 protein-coding DEGs. In the TC, a total of 41 local network clusters were found. The top 10 network clusters showed a significant functional correlation among DEGs involved in synaptic functionality, and these were mainly related to the regulation of both glutamatergic and GABAergic synapses (Supplementary Table S5). A similar outcome was obtained in the functional enrichment analysis when considering the top five STRING GO terms for biological processes, molecular functions, and cellular components (Supplementary Table S6), which was consistent with the GO analyses performed on all DEGs. Interestingly, the considered DEGs were downregulated in the TC of AD subjects, excluding *CFTR*, which was upregulated. *CFTR* encodes for a member of the ATP-binding cassette (ABC) transporter (ATP-Binding Cassette Sub-Family C, Member 7), whose expression, which is characteristic of epithelial tissues, was also observed in the human brain [26].

#### 2.1.3. Parietal Cortex—PC

**Figure 3:**
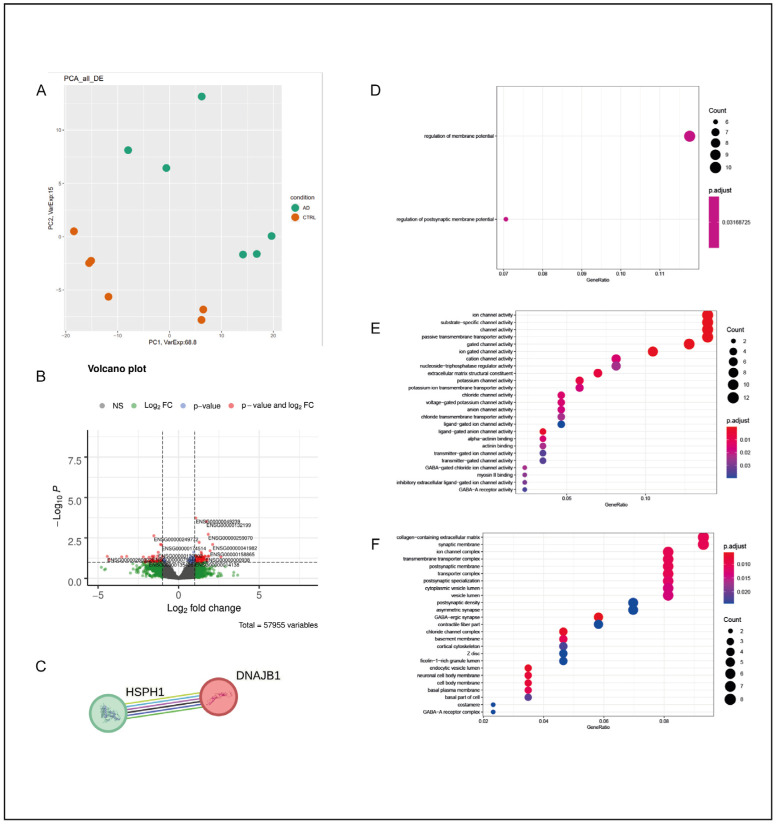
Parietal cortex. (**A**) PCA of DEGs in the PC of AD subjects in comparison with CTRL subjects. In this case, the separation between AD subjects and CTRL subjects was less defined, suggesting a minor difference between the two groups from a statistical point of view. (**B**) Volcano plot of DEGs between AD and CTRL subjects. Genes were plotted in order to emphasize both statistical significance (*p*-value) and the magnitude of change (Log_2_ fold change). Genes whose deregulation was most statistically significant and those with a large fold change are represented by red dots. (**C**) Interaction network for the PC obtained through STRING. The two nodes are represented by *HSPH1* and *DNAJB1*. Interactions between the two nodes were determined using curated datasets and experimental determinations. (**D**) GO-enriched terms in the PC of AD vs. CTRL for biological processes, molecular functions (**E**), and cellular components (**F**). The dots in each category may vary in color and dimension; a color that tends toward red indicates a greater statistical significance, while large dots indicate a higher number of genes of that specific enrichment term that were deregulated in the analyzed sample in comparison with the original GO classification. In this case, the reduced number of DEGs observed in the PC resulted in a less defined enrichment analysis with a minor degree of statistical significance.

In the PC of AD subjects, the total number of DEGs was 109, with the majority of them being protein-coding (92) and upregulated (54). Their classification is reported in Table 1. The PCA division based on the expression levels of all deregulated mRNAs and lncRNAs in the PC of AD subjects and CTRL subjects is represented in Figure 3A, and this resulted in a consistent clustering of AD and CTRL subjects.

The GO biological process analysis resulted in the “regulation of membrane potential” and the “regulation of postsynaptic membrane potential” (Figure 3D), with the majority of deregulated biological activities (Figure 3E) being related to the functionality of ion and gated channels. Therefore, the involvement of cellular components (Figure 3F) that were primarily related to cellular membranes was observed. We then used the biological STRING database to obtain information about protein–protein interactions and perform a functional enrichment analysis in the PC. The number of considered protein-coding DEGs decreased from 92 to 27, and only one interaction network was found between *HSPH1* and *DNAJB1* (Figure 3C). Both *HSPH1* and *DNAJB1* encode for members of two different families of heat shock proteins (HSPs)—the Hsp70 protein family and the DnaJ or Hsp40 protein family [27,28]. Both *HSPH1* and *DNAJB1* were downregulated in the PC of our AD subjects compared with the CTRL subjects. On the other hand, no significant functional enrichment data were obtained with the above-mentioned DEG selection criteria.

#### 2.1.4. Cingulate Gyrus—CG

**Figure 4:**
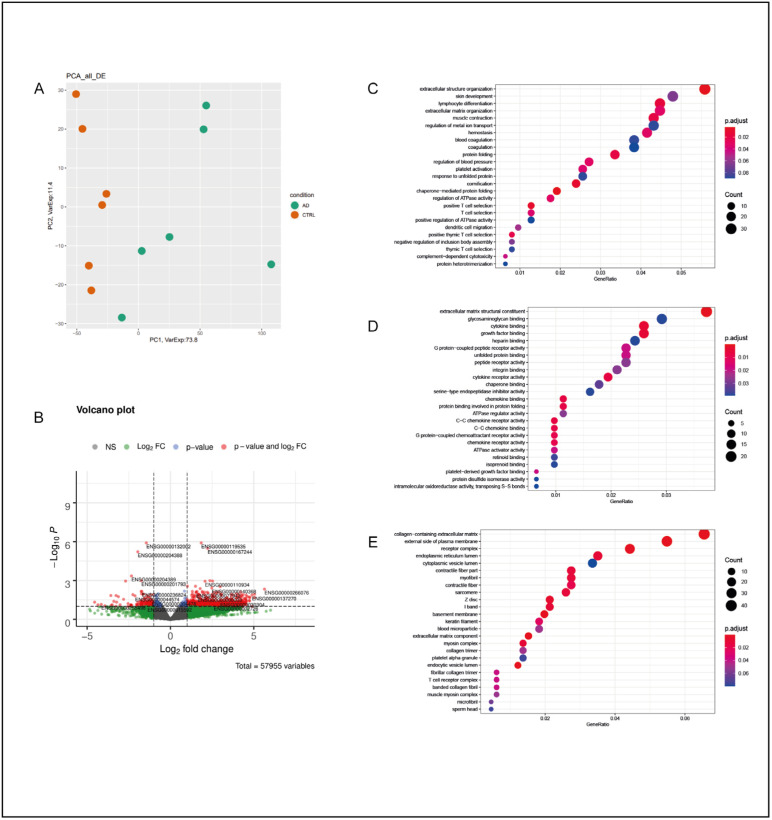
Cingulate gyrus. (**A**) PCA of DEGs in the CG of AD subjects compared with the CTRL subjects. In this case, the separation between AD subjects and CTRL subjects was less defined, suggesting a minor difference between the two groups from a statistical point of view. (**B**) Volcano plot of DEGs between the AD and CTRL subjects. Genes were plotted in order to emphasize both statistical significance (*p*-value) and the magnitude of change (Log_2_ fold change). Genes whose deregulation was most statistically significant and those that had a large fold change are represented by red dots. (**C**) GO-enriched terms in the CG of AD vs. CTRL for biological processes, molecular functions (**D**), and cellular components (**E**). Dots in each category may vary in color and dimension; a color that tends toward red indicates a higher statistical significance, while large dots indicate a higher number of genes of that specific enrichment term that were deregulated in the analyzed sample in comparison with the original GO classification.

In the CG of AD subjects, the total number of DEGs was 1210, with the majority of them being protein-coding (727) and upregulated (617). Their classification is reported in Table 1. The PCA division based on the expression levels of all deregulated mRNAs and lncRNAs in the CG of AD subjects and CTRL subjects is represented in Figure 4A.

For the CG of AD subjects, the GO biological process analysis resulted in a significant deregulation of processes related to protein folding, such as “protein folding”, “response to unfolded protein”, and “chaperone-mediated protein folding” (Figure 4C). Thus, a deregulation of the response to extracellular stimuli was observed, with an alteration in unfolded protein binding and chaperone binding. Moreover, an alteration in cell-signaling-related functions was also observed, and there was involvement of cytokine and chemokine receptor activity (GO molecular function analysis) (Figure 4D), the external side of plasma membranes, and extracellular matrix components (GO cellular component analysis) (Figure 4E). A STRING analysis was performed on 202 out of 727 protein-coding DEGs. In the CG, a total of eight local network clusters were found. This analysis showed a functional correlation between the DEGs involved in several protein-folding-related processes, which were mainly chaperone-related, and in extracellular matrix organization (Supplementary Table S7). A similar outcome was obtained in the functional enrichment analysis when considering the top five STRING GO terms for biological processes, molecular functions, and cellular components (Supplementary Table S8), and the results were consistent with those of the GO analyses performed on all DEGs. The number of DEGs of the top five STRING GO terms in the functional enrichment analysis was 50. Moreover, 16 out of the 50 DEGs were downregulated, while 34 were upregulated. Considering the 16 downregulated genes, 8 of them (*DNAJB1*, *HSPA1B*, *HSPA1A*, *HSPA5*, *DNAJB4*, *HSP90B1*, *HSP90AA1*, and *HSP90AB1*) encode for heat shock protein family members [29], while five of them (*FKBP4*, *PDIA4*, *PDIA2*, *CHORDC1*, and *STIP1*) encode for proteins related to protein folding and trafficking [30,31,32,33]. Interestingly, the remaining three downregulated genes (*CACNA2D1*, *CRELD1*, and *HBB*) are not related to protein folding and trafficking processes.

#### 2.1.5. Substantia Nigra—SN

**Figure 5:**
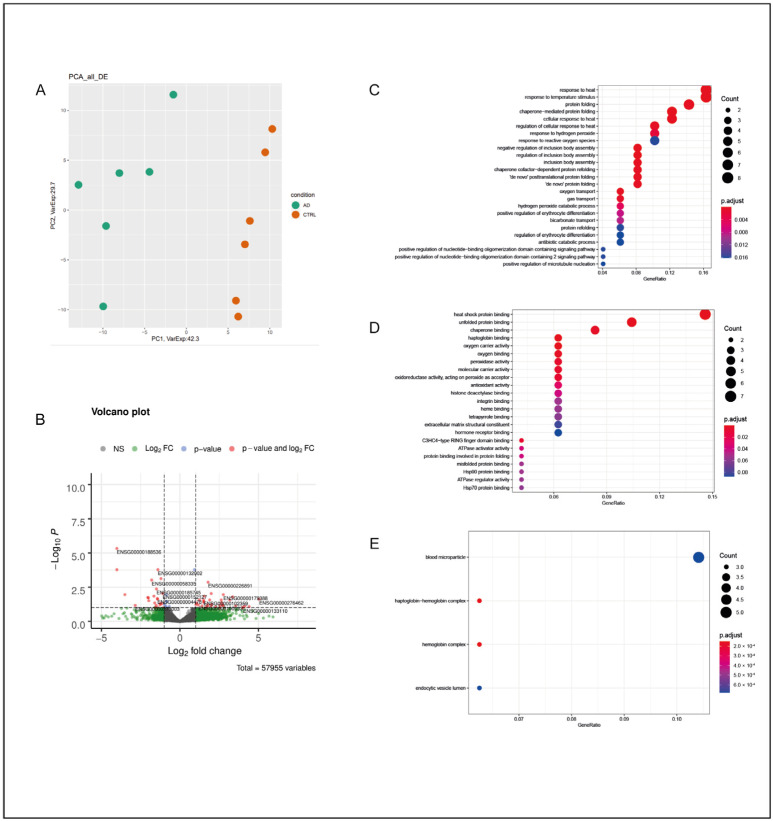
Substantia nigra. (**A**) PCA of DEGs in the SN of AD subjects in comparison with CTRL subjects. AD subjects and CTRL subjects were separated into two distinct groups, suggesting a substantial difference between the two groups from a statistical point of view. (**B**) Volcano plot of DEGs between the AD and CTRL subjects. Genes were plotted in order to emphasize both statistical significance (*p*-value) and the magnitude of change (Log_2_ fold change). Genes whose deregulation was most statistically significant and those that had a large fold change are represented by red dots. (**C**) GO-enriched terms in the SN of AD vs. CTRL for biological processes, molecular functions (**D**), and cellular components (**E**). Dots in each category may vary in color and dimension; a color that tends toward red indicates a higher statistical significance, while large dots indicate a higher number of genes of that specific enrichment term that were deregulated in the analyzed sample in comparison with the original GO classification.

In the SN of the AD subjects, the total number of DEGs was 60, with the majority of them being protein-coding (53) and upregulated (29). Their classification is reported in Table 1. The PCA division based on the expression levels of all deregulated mRNAs and lncRNAs in the AD and NOLD is represented in Figure 5A.

For the SN of AD subjects, the GO biological process analysis resulted in a significant deregulation of processes related to protein folding and chaperone-mediated protein folding. Thus, a strong deregulation of heat shock proteins and chaperone functions was observed (GO molecular function). On the other hand, the GO cellular component analysis revealed a deregulation of genes related to hematic components (Figure 5C–E). A STRING analysis was performed on 30 out of 53 protein-coding DEGs. In the SN, a total of five local network clusters were found, showing a functional correlation between DEGs involved in denatured protein binding and folding and those involved in the haptoglobin–hemoglobin complex (Supplementary Table S9). This result was consistent with the top five STRING GO terms for biological processes, molecular functions, and cellular components in the functional enrichment analysis, which was statistically significant even when considering the small number of selected DEGs (Supplementary Table S10). The DEGs of the top five STRING GO terms in the functional enrichment analysis were all downregulated in the SN of AD subjects. Interestingly, the most downregulated genes (log2(AD subjects/CTRL subjects) ≤ −4) were *HBA2* and *HBB*.

### 2.2. Brain Areas in the AD Cluster According to the Deregulation of the Same Class of Enrichment Terms

**Figure 6:**
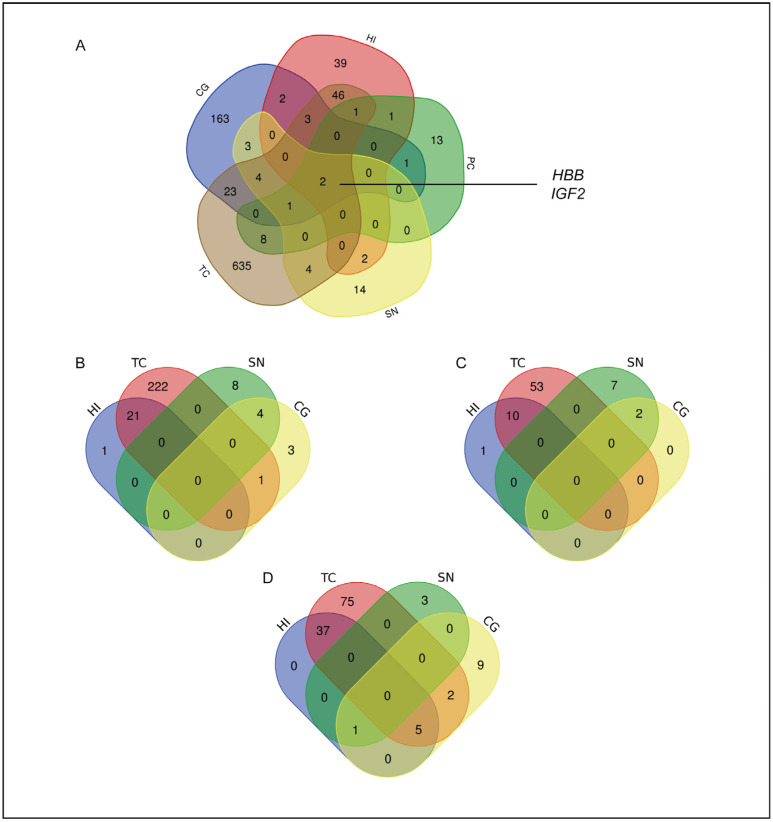
Brain areas of AD subjects were clustered according to the deregulation of the same class of enrichment terms. (**A**) Venn diagram of DEGs across the analyzed brain areas in AD subjects. The selection of DEGs was made by considering protein-coding genes with an adjusted *p* value of ≤0.05. The Venn diagrams referring to the overlapping of GO enrichment terms resulted from the STRING functional enrichment analysis: (**B**) GO biological process terms; (**C**) GO molecular function terms; and (**D**) GO cellular component terms.

All of the investigated brain areas of AD subjects shared the deregulation of two specific genes, *HBB* and *IGF2*. As seen in Figure 6A, in our study, these were both downregulated in all of the analyzed areas. The downregulation of *HBB* in all of the selected areas was validated through qPCR (Supplementary Figure S2).

In addition to the simple overlapping in gene expression profiles, it was possible to observe a similarity in the STRING functional enrichment analysis results between specific brain areas. Interestingly, the GO enrichment terms for biological processes and molecular functions were spontaneously clustered in two different groups of brain areas: the HI and TC, with 21 shared GO terms for biological processes and 10 shared GO terms for molecular functions, as well as the SN and CG, with four shared GO terms for biological processes and two for molecular functions. On the other hand, the grouping of GO enrichment terms for cellular components was less defined. Once again, there was an almost exclusive overlapping between the HI and TC but not for the SN and CG, with an overlapping of deregulated GO cellular component terms that affected the CG, HI, TC, and SN (Figure 6B–D), which was a sign that even if there was a marked difference between areas in terms of biological functionality, the involved cellular components remained almost unaltered.

Therefore, there was a common specialization between the HI and TC and between the CG and SN. In particular, the 21 GO terms for biological processes shared by the HI and TC were related to synaptic functionality; thus, the 10 GO terms for molecular functions were mainly related to the activity of potassium channels and voltage-gated channels (Supplementary Table S11). On the other hand, the four GO terms for biological processes and the two GO terms for molecular functions shared by the SN and CG were related to protein folding and chaperone activity (Supplementary Table S12). No data were reported for the PC due to the absence of significant STRING functional enrichment analysis results. However, it should be noted that the only interaction network found in the PC comprised *HSPH1* and *DNAJB1*, two genes encoding for proteins involved in protein-folding-related processes, as previously reported. Moreover, considering the top five shared GO terms over the two groups of areas, it was possible to observe that the involved genes were all downregulated in AD subjects in comparison with the CTRL subjects.

#### 2.2.1. The HI and TC in AD Are Subject to a Ca^2+^-Related Synaptic Failure with Major Involvement of AZs

The top five GO terms in the STRING functional enrichment analysis for biological processes and molecular functions shared by the HI and TC are reported in Table 2.

The GO:0098693 term includes *BSN*, *CDK5R1*, and *PRKAR1B*. *BSN* encodes for the Bassoon protein, a fundamental zinc-finger protein of AZs, which are the release sites of chemical neurotransmitters [34,35]. Bassoon activity is strictly related to the activity of Piccolo, another zinc-finger protein of AZs that has a link domain for Ca^2+^ and is involved in synaptic vesicle clustering [36]. Furthermore, *PCLO*, the gene encoding for Piccolo, was downregulated in the HI of our AD subjects, together with *ERC2*, a gene encoding for another AZ protein that forms a protein complex with Piccolo and Bassoon and regulates, in particular, the Ca^2+^-dependent release of neurotransmitters [37]. *ERC2* was also downregulated in the TC; *CDK5R1* encodes for p35, the main activator of Cdk5 (cyclin-dependent kinase-5), a key regulator of cytoskeletal remodeling and synaptic activity in the brain parenchyma. Cdk5 is able to inhibit Rac1 signaling through the phosphorylation of RasGRF2, which, in turn, reduces its exchange activity, leading to Rac1 inhibition and the downregulation of the axonal growth process [38]. Rac1 is a Rho family GTPase that regulates actin dynamics to induce axonal growth [39]. Moreover, a downregulation of *RASGRF2*, the gene encoding for RasGRF2, was observed only in the HI of our AD subjects; *PRKAR1B* encodes for a cyclic AMP (cAMP)-dependent protein kinase A (PKA) subunit. Since we observed a downregulation of *PRKAR1B*, it is important to also consider the role of PKA in the neural context. In fact, it was reported that PKA plays a neuroprotective role by modulating several cAMP-dependent processes related to survival mechanisms and synaptic plasticity [40]. *BSN*, *CDK5R1*, and *PRKAR1B* were downregulated in both the HI and TC of our AD subjects. Moreover, we also observed the downregulation of *ADCY1* (GO:0050804, GO:0048167), a gene encoding for the adenylate cyclase 1, which catalyzes the synthesis of cAMP itself in the brain. Notably, ADCY1, as well as the other components of group I of ADCYs (ADCY3 and ADCY8), is activated by Ca^2+^/calmodulin and is five-fold more sensitive to Ca^2+^ than ADCY3 and ADCY8 [41]. Moreover, loss of ADCY1 was associated with impaired synaptic plasticity and deficits in spatial learning [42]. Furthermore, we also observed the shared downregulation of *SLC8A2* and *SYT7* (GO:0050804, GO:0048167). *SLC8A2* encodes for a Na^+^/Ca^2+^ exchanger whose reduced expression was observed in hippocampal CA1 of APP23 and APP-KI mouse models of familial AD, and it was associated with deficits in hippocampus-dependent learning and memory processes [43]. On the other hand, *SYT7* encodes for the presynaptic Ca^2+^ sensor synaptotagmin-7. Synaptotagmin plays a pivotal role in Ca^2+^-dependent synaptic activity, and its expression was also found to be markedly decreased by the combined action of mutated presenilin (PS) and γ-secretase proteolytic activity, which requires amyloid precursor protein (APP) as a substrate, suggesting a role for mutated PS in presynaptic mechanisms [44]. Another Ca^2+^-related protein whose gene expression was downregulated in both the HI and TC of our AD subjects was *NRGN* (GO:0050804, GO:0048167). *NRGN* encodes for the protein neurogranin, which is localized at the post-synaptic terminal and binds calmodulin in the absence of Ca^2+^. Lower *NRGN* expression was associated with higher Aβ and tau pathology in the perirhinal cortex. Moreover, this negative correlation may indicate that the Aβ and tau proteins may contribute to synaptic degeneration in AD through the downregulation of *NRGN* itself [45].

Other genes encoding for synaptic proteins that were downregulated in both the HI and TC were *NPTX1* (Neuronal pentraxin 1, GO:0050804) and *LRRC4* (Leucine-Rich Repeat Containing 4; GO:0050804). The remaining shared DEGs were related to the activity of voltage-gated K^+^ and Na^+^ channels, K^+^-Cl^−^ cotransporter 2, and RNA-binding proteins. Among these genes, the downregulation of *KCNC1* was validated through qPCR in both the HI and LT (Supplementary Figure S3).

#### 2.2.2. Molecular Chaperone Activity Impairment Is a Common Aspect of AD Pathology in the CG and SN

The top five GO terms shared by the CG and SN in the STRING functional enrichment analysis for biological processes and molecular functions are reported in Table 2.

All of the involved DEGs in both the CG and SN of AD subjects were downregulated. The shared DEGs involved in the two GO terms for molecular functions were *DNAJB1*, *HSPA1A*, and *HSPA1B*, and they were also part of all of the GO terms for biological processes. *DNAJB1*, *HSPA1A*, and *HSPA1B* are part of different families of heat shock proteins. Notably, *HSPA1A* and *HSPA1B* encode for two members of the Hsp70 family. Even if Hsp70 family members’ expression decreases with age, induced overexpression of these proteins was associated with a reduction in Aβ peptides in microglial cells and a reduction in tau protein in cell lines and animal models [46,47].

The other DEGs involved in the GO terms for biological processes were *FKBP4*, *CHORDC1*, and *STIP1*. They are part of both the GO:0061077 and the GO:0006457 terms and encode for proteins related to protein folding and trafficking that, however, are not properly molecular chaperones. In particular, *FKBP4* encodes for FKBP Prolyl Isomerase 4, an enzyme that also displays chaperone activity. Its expression was reduced in AD subjects, and it was found to strongly correlate with the aggregation of pathological tau [48]. *STIP1* encodes for the Stress-Induced Phosphoprotein 1, a co-chaperone that modulates the activity of Hsp70 and Hsp90 families’ proteins and is considered as a neuroprotective factor in the context of neurodegeneration. Moreover, in plasma samples from Parkinson’s disease subjects, STIP1-specific autoantibodies were found, suggesting an autoimmune component of the pathology [49]. On the other hand, *CHORDC1* (Cysteine- and Histidine-Rich Domain Containing 1) encodes for a protein involved in centrosome duplication and chaperone-mediated protein folding. Interestingly, a specific methylation profile of *CHORDC1* was found either in the hippocampus or blood of epileptic subjects [50]. Despite this, *CHORDC1* has not been related to AD so far. *HSPA1A* downregulation was validated through qPCR in both the CG and SN (Supplementary Figure S4).

## 3. Discussion

The first objective of this study was to define the “molecular signature” of AD pathology in the brain by comparing the transcriptomic profiles of five different brain regions of AD subjects with those of CTRL subjects. Our second objective was to define a “molecular map” of the spread of AD by overlapping these transcriptomic profiles to highlight common pathways underlying neuropathological changes that are the consequence but not the cause of the disease. This is an original exploratory approach to AD. Indeed, despite using more recent technology with a deeper resolution (single-cell or spatial transcriptomics), previous studies mainly focused on animal models or on a single brain region [51,52]. A recent meta-analysis aimed to explore the differences between transcriptomic signatures across different brain regions in several neurodegenerative diseases, but, again, no information on single regions was reported, and fewer brain regions were analyzed [53].

### Brain Areas Involved in Early and Late Alzheimer’s Disease Show Different Molecular Signatures; Transcriptomic Analysis Highlights Possible Pathogenetic Mechanisms

In all areas, the classification of DEGs suggested a generalized impairment of the related biological processes. In the HI, the deregulation of processes that were mainly related to the synaptic vesicle cycle and synaptic plasticity, together with the involvement of the presynaptic active zone, was observed. We observed a downregulation of all genes except for *LTF*, the gene encoding for Lf. As reported in the reference literature, an increase in the protein level of Lf was observed in AD subjects in comparison with the CTRL subjects [54]. Moreover, a multivariate non-parametric analysis identified Lf as one of the most powerful novel targets in classifying AD subjects from normal old (NOLD) subjects. This, added to the evidence of a strong correlation between Lf secretion from activated microglia and Aβ production, led to the consideration of the inhibition of the interaction between Lf and APP as a potential therapeutic target for AD treatment [55]. In the context of AD pathology, the HI is also known to be prone to early atrophy, an aspect that, together with general cortical gray matter atrophy, is related to the global cognitive impairment that is characteristic of AD subjects [56]. Therefore, it is not surprising that we observe a broader deregulation of GO terms related to synaptic function in the HI of our AD subjects, which was a result that was also in line with previous work in which, in the HI of late-onset AD (LOAD) subjects, deregulation of genes enriched in synapse function, learning, and memory was observed [14].

In the TC, the main outcome was, again, the deregulation of genes related to synaptic functionality; they were all downregulated except for *CFTR*, whose expression, which is characteristic of epithelial tissues, was also observed in the human brain [26]. In the central nervous system (CNS), several ABC transporters mediate the removal of metabolites from the brain interstitial fluid and cerebrospinal fluid (CSF) into peripheral blood. On the other hand, they regulate the passage of potentially toxic circulating compounds into the CNS [57]. In the context of AD, ABC transporters seem to be involved in protecting the CNS against Aβ neurotoxicity through the export of Aβ to the peripheral blood [58]. On the other hand, downregulation of *CFTR* expression was observed in the hypothalamus of AD subjects, suggesting a relationship between *CFTR* downregulation and the reduction in body fat observed in AD pathology [59]. Although the exact role of *CFTR* in physiology and pathology in the CNS remains unclear, it appears to be widely expressed in brain tissue [26].

Concerning the PC, the reduced number of DEGs resulted in only one functional interaction network that was found between *HSPH1* and *DNAJB1*, as both were downregulated in the PC of our AD subjects. The Hsp70 and DnaJ families’ members form oligomers that are involved in protein refolding/degradation. Furthermore, it has been shown that tau fibrils derived from brain tissue can be successfully disaggregated when mixed with DnaJB1 and Hsp70 [29]. Nonetheless, it is important to state once again that, due to the reduced number of DEGs observed in the PC, no significant functional enrichment data were obtained.

In the CG, on the other hand, we observed a deregulation of genes encoding for heat shock protein family members or related to protein folding and trafficking, except for *CACNA2D1*, *CRELD1*, and *HBB*. *CACNA2D1* (Calcium Voltage-Gated Channel Auxiliary Subunit alpha-2/delta-1) encodes for the α_2_δ preprotein, which is post-translationally cleaved to form two polypeptides that are involved in the trafficking and function of voltage-gated calcium Ca_V_1 and Ca_V_2 channels [60]. CaV1 and CaV2 channel family members play a pivotal role in integrating synaptic inputs in neurons and in initiating synaptic transmission at fast synapses, respectively [61]. CRELD1 (Cysteine Rich With EGF-Like Domains 1) encodes for a protein involved in heart development that was recently associated with immune system homeostasis regulation [62]. In particular, low *CRELD1* expression is associated with a low CD4^+^ T cell number in peripheral blood, which is a sign of immunosenescence [63]. Moreover, *CRELD1* acts as an upstream modulator of Wnt signaling, and its upregulation can restore blood–brain barrier dysfunctions due to the disruption of brain endothelial cells that is characteristic of AD [64]. *HBB* (Hemoglobin Subunit Beta) encodes for the adult hemoglobin subunit beta chain, and its expression was also observed in mouse and human neurons [65,66]. As previously reported, the upregulated genes observed in the CG were also involved in the deregulation of several biological processes related to protein folding, but, surprisingly, the most upregulated genes (log2(AD subjects/CTRL subjects) ≥ 4) were *KRT1*, *TYR*, and *ANXA8*. High expression of *KRT1* (Keratin 1) was observed to be protective against oxidative stress in the context of cerebral cavernous malformations [67]. *TYR* encodes for the protein Tyrosinase (Tyr), which is one of the key enzymes in mammalian melanin synthesis, and its expression was also observed in murine brains [68]. Moreover, PS deficiency leads to the aberrant accumulation of Tyr-containing post-Golgi vesicles, a phenomenon that was related to the PS1M146V familial AD mutation; subjects affected by this exhibited a partial loss of function in melanin synthesis and, by extension, Tyr trafficking [69]. *ANXA8* encodes for Annexin A8, a Ca^2+^-regulated phospholipid-binding protein [70] that was found to be involved in long-term potentiation in mice [71]. Other Annexin family members, such as Annexin A1 and Annexin A5, were related to AD pathology. In particular, Annexin 1 was found to be overexpressed in microglia in proximity to Aβ plaques in post-mortem AD brain tissue, suggesting an adaptive response of microglia during inflammation at early stages of the disease [72]. On the other hand, Annexin A5 was found to play a role in reducing neurotoxicity by interacting with amyloidogenic proteins in the context of neurodegenerative diseases and type II diabetes mellitus [73]. These upregulations could express attempts to cope for damage related to neurodegeneration, including oxidative stress, aberrant protein deposition, and inflammation.

Finally, in the SN, the DEG analysis and the functional enrichment analysis resulted in the deregulation of processes related to denatured protein binding and folding and to the haptoglobin–hemoglobin complex. In particular, the most downregulated genes were *HBA2* and *HBB*. *HBA2* encodes for the hemoglobin subunit alpha-2 chain, whose expression was found to be reduced in the context of several neurodegenerative diseases [74]. A similar role is played by HBB, as previously reported. Our second objective was to switch from a region-specific “molecular signature” to a whole-brain “molecular map” of the spread of AD. When overlapping the DEG profiles between the selected regions, the first outcome was the shared deregulation of *HBB* and *IGF2*. As stated above, HBB encodes for the beta polypeptide chain of adult hemoglobin. The decrease or even the absence of hemoglobin expression in neurons and the downregulation of the *HBB* gene are associated with several pathological conditions, including AD [75,76]. In particular, brain hemoglobin is involved in O2 brain homeostasis and the balance of the redox system. In the context of AD pathology, Aβ was observed to induce a molecular response pathway that led to a strong downregulation of the expression of the HBB gene [76]. On the other hand, *IGF2* encodes for insulin-like growth factor 2, a member of the insulin family of polypeptide growth factors that seems to play a pivotal role in brain plasticity, learning, and memory [77]. In addition, its administration in mice subjected to several mnemonic tasks led to memory enhancement [78]. Then, we looked at the shared STRING functional enrichment analysis terms, and we found that there was a spontaneous clustering of brain regions.

The results obtained for the HI and TC underline the importance of Ca^2+^ homeostasis in the HI and TC in the context of AD and in relation to proper synaptic transmission. In AD, neuronal loss is prominent in the cortex and hippocampus [79], in which one of the main signs observed in both in vivo and post-mortem conditions is synaptic loss, an aspect that is more tightly correlated with cognitive impairment than either Aβ deposition or tangle counts [80,81]. All of these aspects also highlighted the possibility of searching for synaptic biomarkers in the CSF of AD patients, a research line that led to encouraging results, such as the identification of higher levels of SNAP-25 and SYT1 in the CSF of AD and prodromal AD cases [82,83].

The transcriptomic signatures of the PC, CG, and SN, on the other hand, could be explained by the fact that loss of proteostasis is also a hallmark of aging [84]. Proteostasis involves mechanisms for the stabilization of correctly folded proteins, which are mainly dependent on the activity of Hsp family proteins, as well as mechanisms for protein degradation [85,86]. Thus, in this pathological context, a positive modulation of these mechanisms could have a neuroprotective effect.

Interestingly, even though the accumulation of Aβ aggregates spread throughout the brain in combination with AD progression, this modulation was absent in the SN of AD subjects, where the expression of molecular chaperones was observed to be downregulated, suggesting that the neuroprotective mechanism against protein misfolding either was not requested or was already compromised in the SN [87]. Notably, we observed a similar pattern in both the SN and CG, in which a low or absent AD pathology was found. This fact suggested that areas with a similar pathological stage may share similar deregulated pathways.

Considering all of these findings, we could make some assumptions in order to explain the pattern of the spread of AD that we observed. 1. We investigated the variations in the transcriptomic profiles of different brain regions in AD using post-mortem brain tissue samples from elderly subjects. 2. The spread of AD starts from the hippocampus and temporal lobe and leads, in its final stage, to the involvement of the whole brain [7]. 3. We observed that there was a clear and “spontaneous” clustering of deregulated GO terms for biological processes and molecular functions between different areas. In particular, the downregulation of DEGs related to the loss of proteostasis was only localized in the CG and SN of our AD subjects. Therefore, we can assume that the loss of proteostasis could be an early aspect of AD pathology that occurs when a specific brain region starts to be affected; this result is also in line with another study highlighting that there are significant differences in gene expression over the course of AD progression, at least for TC, ultimately leading to cognitive impairment [88]. At an early stage, then, the HI and TC also could have shown a similar molecular signature related to the loss of proteostasis, which eventually switched to the deregulation of biological processes related to synaptic transmission. Considering that cognitive impairment is not significantly related to Aβ deposition and, instead, correlates with pTau pathology and synaptic transmission impairment [7,89], we can also assume that the switch from the loss of proteostasis to synaptic impairment occurs when a significant number of unfolded or misfolded proteins are reached, regardless of Aβ deposition, and this could be a significant hallmark of the beginning of pathology.

Limitations: We worked on a small number of subjects. Nonetheless, using post-mortem samples of human brain tissue remains the gold standard for studying brain disorders [90]. We should also consider that the selected subjects were not free from comorbidities, as shown in Table 3. This could also explain the sub-clustering of the subjects in the reported PCAs. On the other hand, this awareness granted us the opportunity to perform the case selection as carefully as possible. Finally, we obtained no information about the roles of specific cell populations and tissue alterations in transcriptomes since no single-cell sequencing analyses or spatial transcriptomic analyses were performed.

Indeed, our results, while consistent with the reference literature, provide new observations that are useful for further research that investigates the relationships between brain areas in brains with increasing neuropathological AD severity.

## 4. Materials and Methods

### 4.1. Clinical and Neuropathological Assessments

All clinical assessments were performed by the same team (neurologists, geriatricians, and neuropsychologists) according to the Abbiategrasso Brain Bank (ABB) protocol [91]. The ABB autopsy and sampling protocol was approved by the Ethics Committee of the University of Pavia on 6 October 2009 (committee report 3/2009) in the context of the InveCeAb study (A Longitudinal Multidimensional Population Study on Brain Aging (InveCeAb), ClinicalTrials.gov, NCT01345110). This study’s procedures were in accordance with the principles outlined in the Declaration of Helsinki of 1964 and the following amendments. All subjects were asked to sign an exhaustive consent form, and the signature of a guardian or the next of kin was requested in the case of non-compos mentis subjects. This study was also supervised by “Associazione Alzheimer Italia”. During the longitudinal observation, brain donors were subjected to a neuromotor and neurocognitive evaluation that included global cognition and a complete neuropsychological assessment of specific cognitive domains (memory, attention, executive functions, language, and visuospatial abilities) according to the DSM-V. The Clinical Dementia Rating (CDR) was used for a synthetic definition of dementia severity [92].

After death, a brain-harvesting procedure was performed within 24 h, and the brain was processed according to the above-mentioned protocol [91]. Briefly, both hemispheres were cut fresh into slices that were alternately frozen or fixed in 10% phosphate-buffered formalin solution. Then, the samples were processed for paraffin inclusion and cut into 8 μm thick serial sections. All neocortical areas and the basal ganglia (BG), hippocampus (HI), cerebellum, and brainstem (SN, pons, and medulla oblongata) were analyzed to carry out a detailed neuropathological characterization with the following stainings and immunohistochemical reactions: hematoxylin and eosin, Cresyl Violet, Luxol Fast Blue, Gallyas, antibodies against amyloids (4G8; BioLegend (San Diego, CA, USA) Cat# 800722, RRID:AB_2861076), phosphoTau (pTau—AT8; Thermo Fisher Scientific (Waltham, MA, USA) Cat# MN1020, RRID:AB_223647), alpha-synuclein (α-SYN—KM51; Leica Biosystems (Nussloch, Germany) Cat# NCL-L-ASYN, RRID:AB_442103), and phospho-TDP-43 (pS409/410-2; Cosmo Bio (Tokyo, Japan) Cat# CAC-TIP-PTD-P02, RRID:AB_1961898) (Supplementary Table S1). Representative pathological pictures of the HI of 3 different subjects with different AD scores (no AD; intermediate AD; high AD) are shown in Supplementary Figure S1.

For this study, six subjects with a neuropathological diagnosis of AD (AD scoring: intermediate AD, high AD) and three CTRL subjects in duplicate (no dementia) were selected. Sample 1 was classified as CTRL despite CDR = 0.5, since the patient’s minimal cognitive disorder was presumably due to the presence of some degree of small-vessel disease in the absolute absence of neurodegenerative burden. The population age ranged from 71 to 89 years old. The particular characteristics of the selected subjects are reported in Table 3.

### 4.2. Transcriptome Profiling

#### 4.2.1. Tissue Sampling and Total RNA Extraction

For this study, 5 brain areas were selected to perform a whole-transcriptome analysis: the temporal cortex (TC), parietal cortex (PC), cingulate gyrus (CG), hippocampus (HI), and substantia nigra (SN). First, 15 mg of cryopreserved brain tissue sample was obtained and homogenized using the CK14 Precellys Lysing Kit (Bertin Technologies, Aix-en-Provence, France). Then, total RNA was extracted using TRIzol Reagent (Sigma Aldrich, Milan, Italy) according to the manufacturer’s instructions. Quantification of the extracted RNA was performed using a NanoDrop™ One/OneC Microvolume UV-Vis spectrophotometer (Thermo Fisher Scientific, Waltham, MA, USA, RRID:SCR_023005), and the quality and integrity of the extracted RNA were assessed using a 2200 TapeStation Instrument (RRID:SCR_014994) and an RNA ScreenTape Kit (Agilent, Waldbronn, Germany).

#### 4.2.2. Preparation of Libraries for RNA-Seq

Libraries for RNA-seq from the selected brain areas were prepared with the CORALL Total RNA-seq Library Prep Kit (Lexogen, Wien, Austria) using 500 ng of total RNA according to the manufacturer’s instructions. The quality of the libraries was then analyzed using a Tape Station system (D1000 High-Sensitivity Kit, Agilent, Waldbronn, Germany) and quantified using a Qubit™ dsDNA HS Assay Kit (Invitrogen, Waltham, MA, USA). The sequencing was performed with a NextSeq 500/550 High Output v2.5 kit (150 cycles, Illumina, San Diego, CA, USA; Illumina NextSeq 550 System, RRID:SCR_016381).

#### 4.2.3. Quantitative PCR (qPCR)

Using human gene sequences available from the NCBI (www.ncbi.nlm.nih.gov/nucleotide, accessed on 15 January 2023), PCR oligonucleotides for gene pairs were selected using Primer3plus online (https://www.bioinformatics.nl/cgi-bin/primer3plus/primer3plus.cgi, accessed on 20 January 2023) (Supplementary Table S2). Then, 500 ng of RNA was reverse transcribed using the iScript™ cDNA Synthesis Kit (BioRad, Segrate, Italy). Quantitative PCR amplification was performed using SYBR Green Master Mix (BioRad, Segrate, Italy) and 25 ng of cDNA. The reaction was performed using the CFX Connect™ Real-Time PCR Detection System (BioRad, Segrate, Italy, RRID:SCR_018064). Cycle threshold (Ct) values were automatically recorded for each replicate qPCR reaction, and the mean Ct values were normalized against those determined for GAPDH. Fold-expression differences were determined using the 2-ΔΔCt method.

#### 4.2.4. Bioinformatic Data Analysis

FastQ files were generated via Illumina bcl2fastq2, starting from raw sequencing reads produced by an Illumina NextSeq sequencer (Version 2.17.1.14, http://support.illumina.com, accessed on 4 October 2022). Gene and transcript intensities were computed using the STAR/RSEM software with GRCh38 (STAR 2.7.10b: RSEM v 1.3.3 Gencode Release 27) as a reference while using the “stranded” option. Differential expression analysis was performed using the R package DESeq.2. DEGs, which were grouped as coding and non-coding genes, were considered differentially expressed and retained for further analysis with |log2(AD subjects/CTRL subjects)| ≥ 1 and a false discovery rate (FDR) of ≤0.1, considering the CTRL subjects as a reference. qPCR was conducted for the technical validation of RNA-seq.

#### 4.2.5. Gene Set Enrichment Analysis (GSEA) and Statistical Analysis

GSEA was performed on the coding genes. Pathway analysis was performed using KEGG pathway analysis (Kyoto Encyclopedia of Genes and Genomes, http://www.genome.ad.jp/KEGG, accessed on 29 November 2022), WikiPathways analysis, and Gene Ontology (GO) for the biological processes, cellular components, and molecular functions of differentially expressed genes via the enrichR web tool [93]. R software (R software (Version 2023.09.1+494. pro2)) was used to generate Dotplot graphs (using the ggplot2 library). For the functional enrichment analysis, the STRING database (https://string-db.org/, accessed on 10 October 2023) was used, and only protein-coding DEGs with an adjusted *p* value of ≤0.05 were selected. To improve statistical significance and increase the stringency of the selection, a minimum required interaction score with a high level of confidence (0.700) was set. The statistical significance of local network clusters and GO terms was defined with the “strength” and “false discovery rate” values. Venn diagrams were obtained using a specific web tool (https://bioinformatics.psb.ugent.be/webtools/Venn/, accessed on 20 October 2023). For the qPCR results, figures and comparisons of fold-expression differences (2-ΔΔCt method) between brain areas were obtained with RStudio Desktop (Version 2023.09.1+494.pro2, Posit Software, Boston, MA, USA). For comparisons between two areas, the Mann–Whitney test was performed; for comparisons between more than two areas, the Kruskal–Wallis test with Dunn’s post hoc test and Benjamini–Hochberg correction was performed. Data with *p* values of ≤0.05 were considered statistically significant.

## 5. Conclusions

With this work, we were able to observe the transcriptomic signature of AD pathology in different areas of the post-mortem human brain. We then had the opportunity to explore the molecular basis of AD and speculate on the etiology of the disease, reporting that different brain areas have different gene expression profiles. In particular, we observed that the HI and TC are affected by synaptic transmission impairment, while the CG and SN, as well as the PC in a non-statistically significant way, are characterized by a loss of proteostasis. This led us to assume that the spread of AD may follow a time- and severity-related pattern that arises from the age-dependent loss of proteostasis and leads to synaptic impairment.

All things considered, our step in this direction highlights the transcriptomic differences between AD brain areas and the identification of novel biological targets that could be useful for the development of therapeutic approaches. However, further experiments are required in order to confirm this hypothesis and potentially investigate what happens in relation to the severity of AD pathology.

## Figures and Tables

**Table 1:** Number of statistically significant differentially expressed genes in the HI, TC, PC, CG, and SN in terms of upregulated transcripts, downregulated transcripts, and total transcript count.

Brain Regions	Total Count	mRNA		lncRNA	
		Upregulated	Downregulated	Upregulated	Downregulated
HI	206	67	110	12	17
TC	1571	371	781	344	75
PC	109	54	38	10	7
CG	1210	617	110	436	47
SN	60	29	24	6	1

**Table 2:** Classification of the GO terms for biological processes and molecular functions, with the related DEGs, shared by the group of brain areas consisting of the HI and TC and the group of brain areas consisting of the CG and SN.

Brain Regions	GO Aspects	GO Unique Identifier	GO Term Name	Shared DEGs
**HI, TC**	Biological processes	GO:0098693	Regulation of synaptic vesicle cycle	*BSN*, *CDK5R1*, *PRKAR1B*
		GO:0001508	Action potential	*SCN2B*, *KCNB1*, *KCNIP2*, *KCNC2*, *KCNA2*
		GO:0048167	Regulation of synaptic plasticity	*SLC8A2*, *CPEB3*, *NRGN*, *ADCY1*, *KCNB1*, *PRKAR1B*, *SYT7*
		GO:0050804	Modulation of chemical synaptic transmission	*SLC8A2*, *LRRC4*, *CPEB3*, *NRGN*, *ADCY1*, *NPTX1*, *KCNB1*, *PRKAR1B*, *CELF4*, *SYT7*
		GO:0010038	Response to metal ions	*KCNC1*, *ADCY1*, *NPTX1*, *KCNB1*, *KCNIP2*, *DMTN*, *SYT7*, *KCNC2*
	Molecular functions	GO:0005251	Delayed rectifier potassium channel activity	*KCNC1*, *KCNB1*, *KCNC2*, *KCNA2*
		GO:0005249	Voltage-gated potassium channel activity	*KCNC1*, *SCN2B*, *KCNB1*, *KCNV1*, *KCNC2*, *KCNA2*
		GO:0005267	Potassium channel activity	*KCNC1*, *SCN2B*, *KCNB1*, *KCNIP2*, *KCNV1*, *KCNC2*, *KCNA2*
		GO:0015079	Potassium ion transmembrane transporter activity	*KCNC1*, *SCN2B*, *KCNB1*, *SLC12A5*, *KCNIP2*, *KCNV1*, *KCNC2*, *KCNA2*
		GO: 0022843	Voltage-gated cation channel activity	*KCNC1*, *SCN2B*, *CACNA1E*, *KCNB1*, *KCNV1*, *KCNC2*, *KCNA2*
**CG, SN**	Biological processes	GO:0051085	Chaperone cofactor-dependent protein refolding	*DNAJB1*, *HSPA1B*, *HSPA1A*
		GO:0061077	Chaperone-mediated protein folding	*FKBP4*, *DNAJB1*, *CHORDC1*, *HSPA1B*, *HSPA1A*
		GO:0006457	Protein folding	*FKBP4*, *DNAJB1*, *CHORDC1*, *STIP1*, *HSPA1B*, *HSPA1A*
		GO:0090084	Negative regulation of inclusion body assembly	*DNAJB1*, *HSPA1B*, *HSPA1A*
	Molecular functions	GO:0044183	Protein folding chaperone	*DNAJB1*, *HSPA1B*, *HSPA1A*
		GO:0051082	Unfolded protein binding	*DNAJB1*, *HSPA1B*, *HSPA1A*

**Table 3:** Characteristics of the selected subjects. Legend: CDR = Clinical Dementia Rating scale; AD = Alzheimer’s disease; F = female; M = male; NOLD = normal old; NCD = neurocognitive disorder; DM2 = diabetes mellitus type 2; CVD = cardiovascular disease; AHT = arterial hypertension; SVD = small-vessel disease; CAA = cerebral amyloid angiopathy: 1 mild; 2 moderate; 3 severe; P = parenchymal; M = meningeal; cap = capillary involvement; LATE = limbic-predominant age-related TDP-43 encephalopathy (TDP-43—TAR DNA-binding protein 43); PMI = post-mortem interval.

Samples	Gender	Comorbidity	ApoE	Cognitive State	MMSE	CDR	Age at Death	Death Cause	Thal Phase	Braak Stage	AD Scoring (Montine)	CAA (Love)	Additive Pathologies	Vascular Pathology (Skrobot)	PMI	Groups
**1**	M	Cirrhosis, CVD	2//3	NOLD	28	0.5	81	cachexia	0	0	no	no	no	low	19h30′	CTRL
**2**	F	Lung cancer	3//3	NOLD	30	0	71	cachexia	0	I	no	no	no	low	16h	CTRL
**3**	M	CVD; DM2	3//3	NOLD	26	0	79	cachexia	1	I	low	no	no	low	3h15′	CTRL
**4**	F	AHT; CVD; DM2	3//4	Major-NCD (AD; SVD)	21	2	82	cachexia	4	III	int	2M	no	moderate	11h	AD
**5**	F	CVD; DM2	2//3	Major-NCD (AD)	0	5	78	arrhythmia	4	IV	high	no	no	low	8h	AD
**6**	M	AHT	3//3	Major NCD (AD)	0	4	80	cachexia	4	V	high	3P; 3M	no	low	15h30′	AD
**7**	F	CVD; CVD	3//3	Major-NCD (AD; SVD)	2	4	85	arrhythmia	5	V	high	2Pcap; 3M	LATE	moderate	15h30′	AD
**8**	F	AHT; CVD	3//4	Major-NCD (AD; SVD)	0	5	89	cachexia	5	V	high	3Pcap; 3M	LATE	moderate	15h20′	AD
**9**	M	no	3//3	Major-NCD (AD)	4	4	80	arrhythmia	5	VI	high	2M	LATE	no	15h	AD

## Data Availability

The datasets generated and/or analyzed during the current study are available in the GEO repository.

## Supplementary Materials

The following supporting information can be downloaded at: https://www.mdpi.com/article/10.3390/ijms252011117/s1.
