# Supplementary material for: A Map of Transcriptomic Signatures of Different Brain Areas in Alzheimer’s Disease
Source: Int J Mol Sci. 2024 Oct 16;25(20):11117. doi: 10.3390/ijms252011117 (PMC11508373; doi:10.3390/ijms252011117)
Supplement: Supplementary file 1 [file ijms-25-11117-s001.zip › Supplementary Figures.pdf]

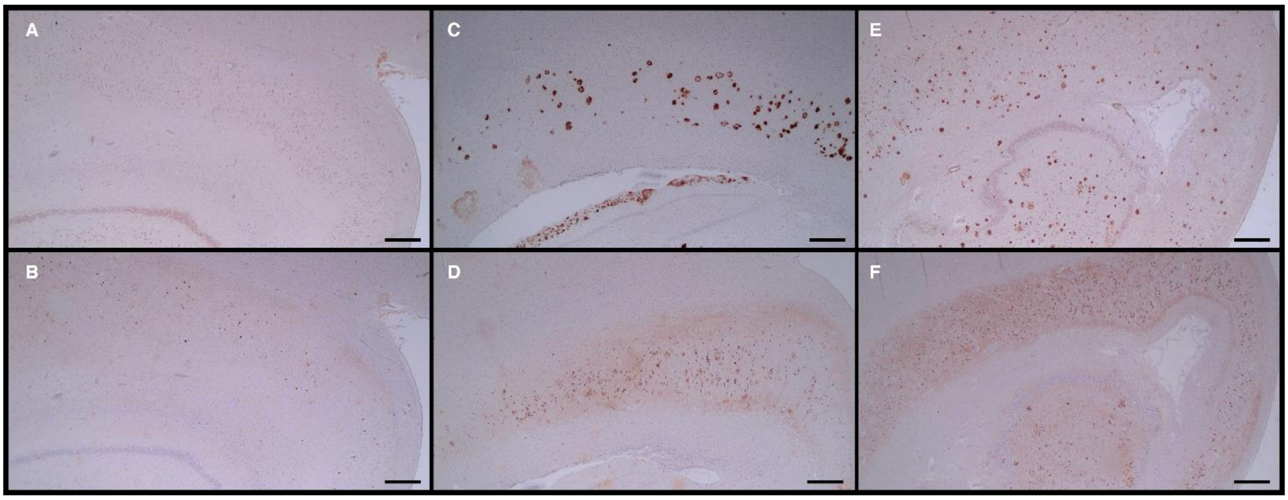

Supplementary Figure S1: Representative images of the hippocampus of 3 out of the 9 subjects, demonstrating different stages of pathology. Panels A and B: neuropathological picture of case n.2 with no AD pathology (Thal 0; Braak I), showing the absence of β-amyloid deposits (A - 4G8 antibody) and a scant presence of neurofibrillary tangles (B - AT8 antibody). Panels C and D: neuropathological picture of case n.4 with intermediate AD pathology (Thal 4; Braak III), showing dense and cored β-amyloid plaques (C - 4G8 antibody), and moderate neurofibrillary tangles and threads with some neuritic plaques (D - AT8 antibody). Panels E and F: neuropathological picture of case n.6 with high AD pathology (Thal 4; Braak V), showing abundant dense and cored β-amyloid plaques (E - 4G8 antibody) and severe neurofibrillary tangles and threads with neuritic plaques (F - AT8 antibody). Images acquired with magnification 2X. Scale bars = 500 µm.

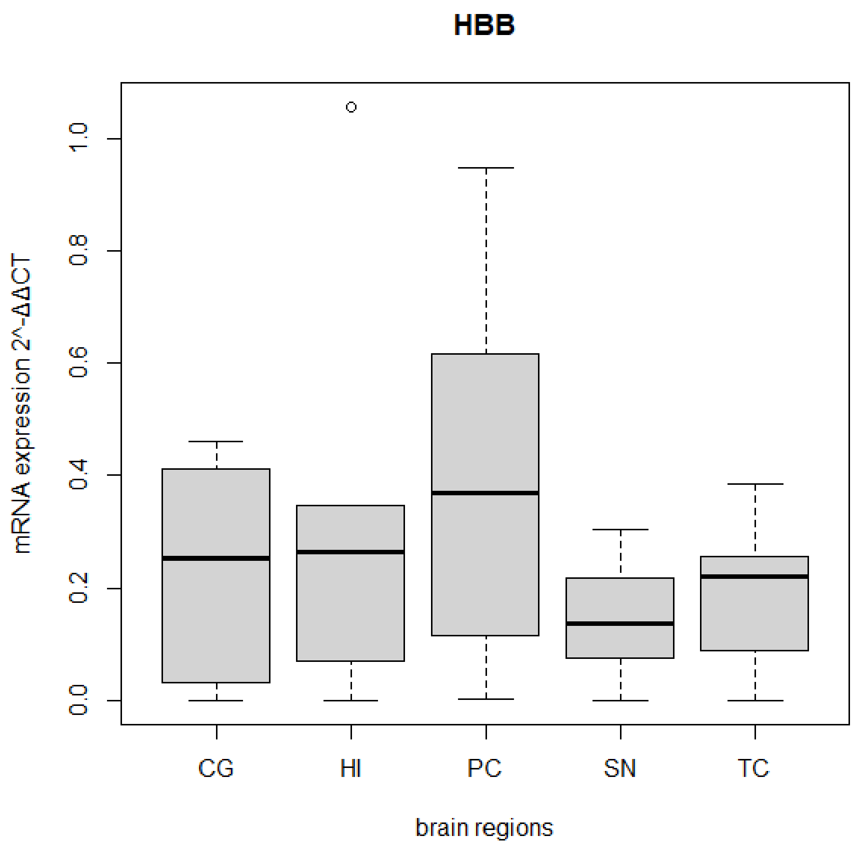

Supplementary Figure S2: Box plot of the relative expression of HBB in CG, HI, PC, SN, TC measured through qPCR. The mRNA expression of HBB in NOLD subjects was 2^-ΔΔCT=1. The downregulation of HBB was validated in all the brain areas. The difference of deregulation between the areas resulted not statistically significant.

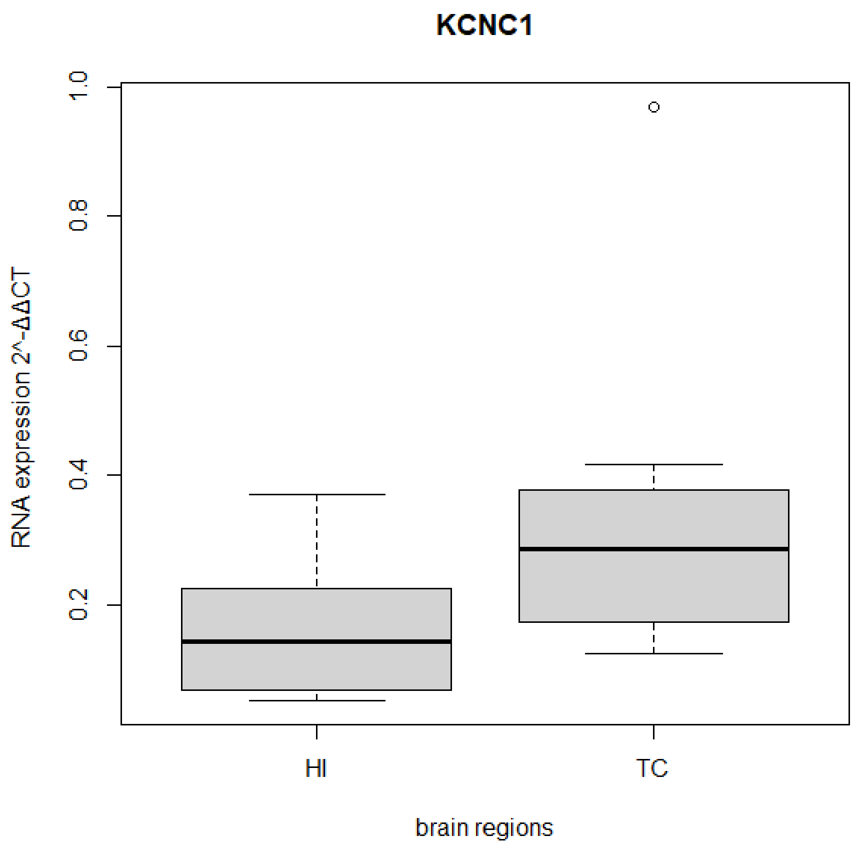

Supplementary Figure S3: Box plot of the relative expression of *KCNC1* in HI and TC measured through qPCR. The mRNA expression of *KCNC1* in NOLD subjects was 2^-ΔΔCT=1. The downregulation of KCNC1 was validated in both HI and LT. Moreover, the difference of deregulation was statistically significant between HI and TC with a major downregulation observed in HI (Mann-Whitney test, *P* value = .0232).

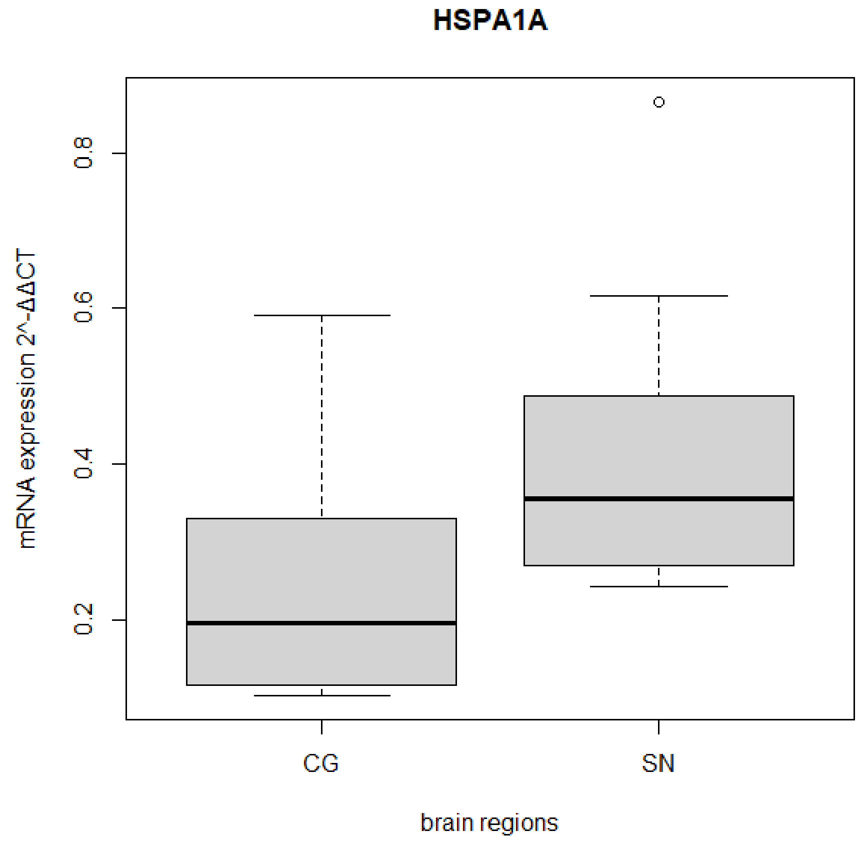

Supplementary Figure S4: Box plot of the relative expression of *HSPA1A* in CG and SN measured through qPCR. The mRNA expression of *HSPA1A* in NOLD subjects was 2^-ΔΔCT=1. The downregulation of HSPA1A was validated in both CG and SN. The difference of deregulation between the areas resulted statistically significant (Mann-Whitney, *P* value = .0262).
